# Determinants of cardiorespiratory fitness in very long-term survivors of allogeneic hematopoietic stem cell transplantation: a national cohort study

**DOI:** 10.1007/s00520-020-05644-1

**Published:** 2020-08-21

**Authors:** Ole Henrik Myrdal, Phoi Phoi Diep, Ellen Ruud, Lorentz Brinch, Richard John Massey, Elisabeth Edvardsen, Johny Kongerud, May B. Lund, Liv Ingunn Sikkeland

**Affiliations:** 1 Department of Respiratory Medicine, Oslo University Hospital, Rikshospitalet, Box 4950 Nydalen, 0424 Oslo, Norway; 2 Institute of Clinical Medicine, University of Oslo, Oslo, Norway; 3 Department of Pediatric Oncology and Haematology, Oslo University Hospital, Rikshospitalet, Oslo, Norway; 4 Department of Pediatric Research, Division of Pediatric and Adolescent Medicine, Oslo University Hospital, Oslo, Norway; 5 Department of Haematology, Oslo University Hospital, Rikshospitalet, Oslo, Norway; 6 Department of Cardiology, Oslo University Hospital, Rikshospitalet, Oslo, Norway; 7 Department of Physical Performance, The Norwegian School of Sport Sciences, Oslo, Norway; 8 Department of Pulmonary Medicine, Oslo University Hospital Ullevål, Oslo, Norway

**Keywords:** Allogeneic hematopoietic stem cell transplantation, Long-term follow-up, Cardiorespiratory fitness, Cardiac function, Pulmonary function

## Abstract

**Purpose:**

Survivors of allogeneic hematopoietic stem cell transplantation (allo-HSCT) are at risk for cardiopulmonary adverse events. Data on long-term effects on cardiorespiratory fitness are limited. To address the gap in knowledge, we aimed to determine peak oxygen uptake (V̇O_2_peak) and identify associations between cardiorespiratory fitness and clinical characteristics, self-reported physical activity, cardiac, and pulmonary function.

**Methods:**

In a nationwide, single-center cross-sectional study, 90 survivors [aged median (range) 35 (17–54) years, 56% females] were examined, 17 (6–26) years after allo-HSCT. Myeloablative conditioning comprised busulfan/cyclophosphamide or cyclophosphamide only. Methods included pulmonary function tests, echocardiography, and cardiopulmonary exercise test.

**Results:**

Chronic graft-versus-host disease (cGVHD) was found in 31% of the subjects, of whom 40% had bronchiolitis obliterans syndrome (BOS). Seventy-one percent of the survivors did not meet WHO recommendations for physical activity and 42% were overweight. Reduced gas diffusion (DL_CO_) and systolic ventricular dysfunction (LVEF) were found in 44% and 31%, respectively. For the group, mean (95% CI), V̇O_2_peak was 36.4 (34.7–38.0) mL/min/kg [89 (85–93)% of predicted]. V̇O_2_peak was low at 43%. Cardiopulmonary factors and deconditioning were equally common limitations for exercise. In a multiple linear regression model, low V̇O_2_peak was associated with low DL_CO_, low LVEF, BOS, overweight, and inactivity.

**Conclusion:**

Half of the survivors had reduced cardiorespiratory fitness median 17 years after allo-HSCT. Cardiopulmonary factors and deconditioning were equally common limitations to exercise. We encourage long-term cardiopulmonary monitoring of allo-HSCT survivors and targeted advice on modifiable lifestyle factors.

**Electronic supplementary material:**

The online version of this article (10.1007/s00520-020-05644-1) contains supplementary material, which is available to authorized users.

## Introduction

Survivors of allogeneic hematopoietic stem cell transplantation (allo-HSCT) are at risk for both cardiac and pulmonary adverse events, caused by the myeloablative conditioning prior to allo-HSCT and the immunological response induced by the hematopoietic stem cell graft after allo-HSCT [1, 2]. In addition, most patients with hematologic malignancies have received chemotherapy with or without radiation before they became candidates for allo-HSCT.

Late-onset non-infectious pulmonary complications (LONIPCs) occur in up to one-fifth after allo-HSCT and comprise leading causes of morbidity and mortality [3]. The most frequent LONIPC is bronchiolitis obliterans syndrome (BOS), which is linked to chronic graft-versus-host disease (cGVHD) [4]. Cardiovascular disease is another cause of morbidity and mortality in long-term allo-HSCT survivors [5]. Risk factors for cardiovascular disease have been reported to be > 4-fold higher in allo-HSCT survivors than in the general population [6], with a clear association between multiple risk factors and subsequent development of cardiovascular disease [2].

Measurement of peak oxygen uptake (V̇O_2_peak) through cardiopulmonary exercise test (CPET) represents the gold standard assessment of cardiorespiratory fitness and is useful to assess the global effect of cardiac and/or pulmonary impairments [7]. CPET also provides information about the risk of cardiovascular disease, all-cause mortality, and cancer mortality [8, 9].

Cardiorespiratory fitness in long-term survivors of allo-HSCT has been scarcely investigated [10–14]. Furthermore, most previous studies have been restricted to children [12, 14] or been limited by small and/or inhomogeneous patient materials [11, 13], and follow-up has not exceeded 10 years [10–14]. In Norway, all allo-HSCTs have been performed at Oslo University Hospital. The patients were recruited from the entire country, referred and selected according to uniform criteria, and subjected to standardized treatment procedures. Today, this single-center policy has provided us with a well-defined national patient population available for follow-up.

In the present study, our primary aim was to determine cardiorespiratory fitness assessed by V̇O_2_peak in a nationwide cohort of young very long-term allo-HSCT survivors, who had received myeloablative conditioning with chemotherapy only and not total body irradiation (TBI). Secondly, we aimed to identify associations between cardiorespiratory fitness and clinical characteristics, self-reported level of physical activity, and cardiac and pulmonary function.

## Methods

### Design and study population

**Fig. 1:**
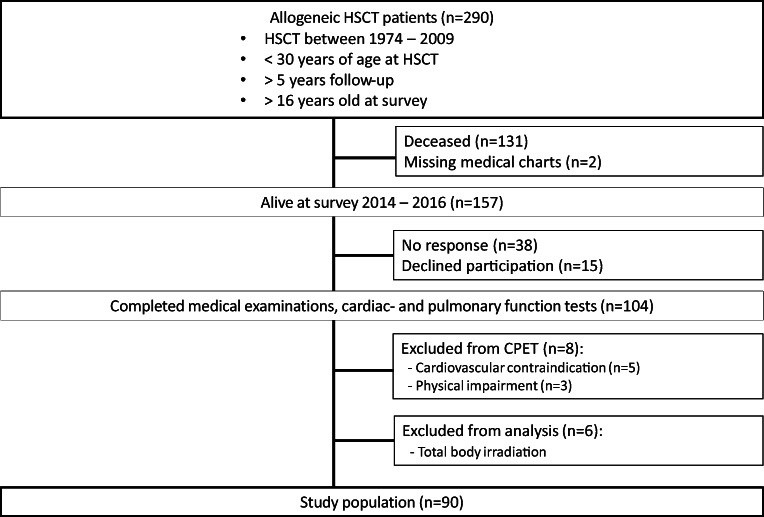
Study selection of long-term survivors after allogeneic HSCT

The study was part of a large, nationwide cross-sectional study covering a broad range of late treatment-related effects, conducted at Oslo University Hospital [15, 16]. All subjects were survivors of allo-HSCT, performed in childhood or early adulthood. Inclusion criteria were age < 30 years at allo-HSCT, > 16 years at the time of the survey, and minimum 5-year follow-up. At the time of the survey, 157 subjects were alive, and 104 subjects participated (Fig. 1). Exclusion criteria in the present study were TBI conditioning (*n* = 6), cardiovascular contraindications (*n* = 5), and physical inability to perform CPET, using wheelchair (*n* = 3). Cardiovascular contraindications included severe, untreated hypertension, coronary fistula to the pulmonary artery, cardiomyopathy, aortic valve stenosis, and significant pulmonary hypertension. In total, 90 subjects aged [median (range) 35 (17–54) years, 56% females] were included. The Regional Committee for Medical and Health Research Ethics (2014/370) approved the study. Written informed consent was obtained from all participants.

### Clinical assessment

At follow-up, medical records were reviewed for treatment data. All participants underwent a medical examination, blood sampling, and a standardized interview. Age, gender, body mass index (BMI, kg/m^2^), smoking habits, physician-diagnosed cardiovascular or pulmonary disease, and current medication were registered (Table 1). Overweight was defined as BMI ≥ 25 kg/m^2^ and obesity BMI ≥ 30 kg/m^2^, according to the World Health Organization (WHO) Classification [17]. The self-reported physical activity level was assessed using the WHO recommendations [18]. The survivors were categorized as meeting the recommendations if their activity included ≥ 150 min/week with moderate-intensity exercise or ≥ 75 min/week with high-intensity exercise. cGVHD was diagnosed according to the National Institute of Health (NIH) criteria [19]. Hypertension was defined as current treatment with antihypertensive agents.

**Table 1:** Characteristics of 90 long-term survivors of allogeneic HSCT

	*n* = 90
Male/female, *n* (%)	40/50 (44/56%)
Age at transplantation, median (range)	20 (0.3–30)
Age at follow-up, median (range)	35 (17–54)
Years of observation, median (range)	17 (6–26)
BMI, kg/m^2^, median (range)	23.8 (15.8–43.8)
Overweight (BMI 25.0–29.9 kg/m^2^), *n* (%)	25 (28%)
Obese (BMI ≥ 30 kg/m^2^), *n* (%)	13 (14%)
Diabetes	2 (2%)
Physician-diagnosed asthma pre-allo-HSCT, *n* (%)	9 (10%)
Smoking	
Never, *n* (%)	61 (68%)
Former, *n* (%)	16 (18%)
Daily, *n* (%)	13 (14%)
Pack-years, median (range)	3 (1–24)
Underlying diagnosis	
Malignancy, *n* (%)	66 (73%)
Acute myeloid leukemia, *n* (%)	30 (33%)
Chronic myeloid leukemia, *n* (%)	20 (22%)
Acute lymphoblastic leukemia, *n* (%)	9 (10%)
Other malignant, *n* (%)	7 (8%)
Severe aplastic anemia, *n* (%)	15 (17%)
Other non-malignant, *n* (%)	9 (10%)
Conditioning	
Cyclophosphamide/busulfan or cyclophosphamide, *n* (%)	88 (98%)
None, *n* (%)	2 (2%)
Donor	
Matched related donor, *n* (%)	56 (62%)
Matched unrelated donor, *n* (%)	27 (30%)
Haploidentical donor, *n* (%)	7 (8%)
Graft-vs-host disease	
Diagnosed earlier	
Acute, grades 0–I, *n* (%)	66 (73%)
Acute, grades II–IV, *n* (%)	24 (27%)
Chronic, limited, and extensive, *n* (%)	35 (39%)
Diagnosed at follow-up	
Chronic, grades 1–3 (NIH criteria), *n* (%)	28 (31%)
Bronchiolitis obliterans syndrome, *n* (%)	11 (11%)

*Allo-HSCT*, allogeneic hematopoietic stem cell transplantation; *BMI*, body mass index

### Blood samples

Fasting blood samples were collected at 8:00 am and analyzed for hemoglobin, lipids, glycated hemoglobin (HbA1c), and N-terminal proB-type natriuretic peptide (proBNP). For Hb and proBNP, the hospital’s reference values for males and females were used (Hb 13.4–17.0 g/dL and 11.7–15.3, and proBNP < 10 pmol/L and < 20 pmol/L in the age group 18–48 years, respectively). Hypercholesterolemia was defined as low-density lipoprotein > 4.1 mmol/L or the use of lipid-lowering agents. Diabetes mellitus was defined as HbA1c > 6.5% or the current use of antidiabetic medication.

### Pulmonary function

Pulmonary function measurements (Jaeger Master Screen Body, Würzburg, Germany) were performed in accordance with the guidelines of ERS [20, 21]. Recorded variables were total lung capacity (TLC), residual volume (RV), forced vital capacity (FVC), forced expiratory volume in 1 s (FEV_1_), and gas diffusion capacity (DL_CO_). The predicted values for FVC and FEV_1_ were taken from the Global Lung Initiative [22] and static lung volumes and DL_CO_ from the European Community for Steel and Coal [23]. Obstructive impairment, i.e., BOS, was diagnosed according to NIH criteria [19]. Restrictive impairment was defined as TLC < 80% of predicted value, and gas diffusing impairment as DL_CO_ < 80% of predicted value, corresponding to the lower 5th percentile of the reference material and in line with ERS recommendations [24].

### Echocardiography

Echocardiography was performed according to international recommendations [25], using E9 scanners from GE (Horten, Norway). All recordings were obtained by one single sonographer and reviewed by a senior cardiologist, both blinded to all clinical data. Left ventricular ejection fraction (LVEF) was assessed by calculating the modified Simson’s method [25]. Left ventricular systolic dysfunction was defined as LVEF < 52% for men and < 54% for women [25].

### Cardiopulmonary exercise test

All CPETs were performed with the subjects walking on a treadmill (TechnoGym Runrace, Forli, Italy) using a modified Balke protocol [26], the same protocol used for the reference population [27]. In short, after a warm-up phase at 3 km/h and a 2% inclination, the inclination was increased by 2% every 60 s up to 20%. Then, the speed was increased by 0.5 km/h every 60 s until exhaustion. During the test, metabolic gas exchange and ventilatory variables were measured continuously breath-by-breath and averaged over 30-s intervals (Vyntus CPX, CareFusion, Hoechberg, Germany) through a two-way breathing mask (2700 series, Hans Rudolph, inc. Shawnee, USA). Calibration of flow and gas concentration was performed before each test. Maximal heart rate (HR_max_) was recorded with a 12-lead ECG (CustoMed, Ottobrunn, Germany). The test was discontinued at exhaustion, reporting Borg scale 18–20 [28], and respiratory exchange ratio (RER) > 1.10. V̇O_2_peak predicted was calculated from the equations of Edvardsen et al. [27]. Low cardiorespiratory fitness was defined as V̇O_2_peak < 85% of predicted, in accordance with ATS/ACCP guidelines [29]. The ventilatory limitation was defined as breathing reserve ≤ 15% or 11 L/min [30], and gas exchange limitation was defined as V_E_/$\dot{\mathrm{V}}$_CO2_ slope ≥ 34 [31]. The cardiac limitation was defined as oxygen pulse < 80% of predicted, HR < 90% of age-predicted, and HR reserve < 15 beats/min in absences of pulmonary limitations [29]. Oxygen pulse was calculated dividing V̇O_2_peak (mL min^−1^) by HR_max_ [29]. Deconditioning was defined as low V̇O_2_peak in absence of cardiac or pulmonary limitations.

### Reference population

The reference population has been described previously [27]. In brief, in a multicenter study involving regional centers throughout Norway, 759 healthy adults successfully completed CPET on a treadmill. In total, 558 subjects (48% females) were within the same age range as the participants in the present study.

### Statistical analysis

Student *t* test or Mann-Whitney *U* test was used, as appropriate, to compare continuous data between groups, and the chi-square test or Fisher’s exact test to compare categorical variables. Univariate and multiple linear regression analyses were used to detect associations between outcome variables and determinants of interest. The independent variables entered the regression models were those hypothesized a priori for biological or clinical reasons or found to be significant at a 20% level by univariate analysis. Due to collinearity, only one variable from the pulmonary function tests and one age-related variable were included in the final model. Pulmonary function in patients with BOS was analyzed separately since the diagnostic criteria for BOS are based on such tests [19]. Since the chemotherapy regimen used in the treatment of malignant blood disorders may affect both cardiac and pulmonary function, we chose to analyze the relevant variables according to chemotherapy prior to allo-HSCT (yes vs no). A two-sided *p* value ≤ 0.05 was considered significant. Standard statistical analyses were performed with SPSS (IBM SPSS, v26).

## Results

Clinical characteristics are outlined in Table 1. In total, 42% of the subjects were overweight, 14% obese. Subjects with overweight were more frequently treated for hypertension (42% vs 21%, *p* = 0.03) and hypercholesterolemia (29% vs 8%, *p* = 0.008) than those with normal weight. Hematological malignancies comprised the underlying diagnosis in 66 (73%) of the patients of whom 41 (46%) had received treatment with chemotherapy regimens prior to allo-HSCT. Patients diagnosed with chronic myeloid leukemia did not receive chemotherapy routinely, in contrast to the other malignant disorders. Myeloablative conditioning with busulfan/cyclophosphamide or cyclophosphamide had been applied in 98% of the subjects. Two patients with severe combined immunodeficiency did not receive a conditioning regimen. cGVHD (grades 1–3) was diagnosed in 28 (31%) of the survivors and was associated with chemotherapy prior to allo-HSCT (*p* = 0.005). Eleven of the 28 patients with cGVHD had developed BOS. The genders were comparable with respect to all clinical characteristics. The non-responders were younger than those who participated in the study [(median, range) 25 (18–53) vs 35 (17–54) years, *p* < 0.001] and comprised more males (70% vs 44%, *p* = 0.01) but were comparable with respect to malignant vs non-malignant disease prior to allo-HSCT. Pulmonary and cardiac functions according to chemotherapy prior to allo-HSCT are summarized in Table 2. For the entire study group, mean lung volumes and DL_CO_ were above 80% predicted; however, 44% had impaired DL_CO_. Patients who had received chemotherapy prior to allo-HSCT had lower DL_CO_ % predicted than those who had not received such treatment [mean (95% CI) 79 (75–84) vs 87 (83–92), *p* = 0.02]. Patients with BOS had increased RV and reduced FEV_1_, FVC, and FEV_1_/FVC, thus confirming that they met the NIH criteria.

**Table 2:** Blood test, pulmonary, and cardiac function of 90 long-term survivors of allo-HSCT according to chemotherapy prior to HSCT (no/yes)

	All	No	Yes	*p* value	BOS*
Pulmonary function	*n* = 90	*n* = 46 (51%)	*n* = 33 (37%)		*n* = 11 (12%)
TLC % predicted	104 (101–106)	105 (101–108)	103 (98–107)	0.71	105 (98–112)
RV % predicted	117 (112–123)	115 (108–122)	120 (111–129)	0.89	157 (145–170)
FVC % predicted	93 (90–96)	96 (92–100)	90 (86–95)	0.36	80 (73–87)
FEV_1_% predicted	88 (84–92)	92 (87–97)	84 (78–90)	0.17	56 (46–66)
FEV_1_/FVC	0.78 (0.75–0.80)	0.79 (0.76–0.83)	0.76 (0.73–0.79)	0.19	0.58 (0.50–0.66)
DL_CO_ % predicted	83 (80–87)	87 (83–92)	79 (75–84)	0.02	85 (74–95)
Cardiac function		*n* = 49 (54%)	*n* = 41 (46%)		
LVEF (%)	55 (54–56)	57 (55–58)	54 (51–56)	0.01	
Left ventricular dysfunction	28 (31%)	6 (25%)	22 (33%)	0.02	
Hypercholesterolemia	15 (17%)	1 (5%)	14 (25%)	0.51	
Hypertension	37 (41%)	6 (27%)	31 (54%)	0.18	
Blood analysis					
proBNP, pmol/L^†^	5.5 (0.7–79.0)	3.9 (0.7–19.0)	9.0 (1.4–79.0)	< 0.001	
CRP, mg/L^†^	1.5 (0.6–14.0)	1.2 (0.6–14.0)	1.7 (0.6–12.0)	0.11	
Hb, g/dl	14.3 (14.1–14.6)	14.3 (14–14.7)	14.3 (13.8–14.7)	0.78	
HbA1c^†^	5.4 (4.2–6.7)	5.3 (5.2–5.4)	5.4 (5.3–5.6)	0.13	

*BOS patients were separated in a column for the pulmonary function values. *HSCT*, hematopoietic stem cell transplantation; *BOS*, bronchiolitis obliterans syndrome; *TLC*, total lung capacity; *RV*, residual volume; *FVC*, forced vital capacity; *FEV*_*1*_, forced expiratory volume-one second; *DL*_*CO*_, diffusing capacity for carbon monoxide; *LVEF*, left ventricular ejection fraction; *proBNP*, pro B-type natriuretic peptide; *CRP*, C-reactive protein; *Hb*, hemoglobin concentration; *HbA1c*, glycated hemoglobin. Data are presented as mean (95% confidence interval), number (%) or † median (range)

As a group, the survivors had mean LVEF above the lower limit. However, left ventricular systolic dysfunction was found in 31% of the subjects and was associated with having received chemotherapy prior to allo-HSCT (*p* = 0.02). The subjects with left ventricular systolic dysfunction also had higher levels of proBNP than those with normal LVEF [median (range) 7.9 (0.8–79.0) vs 4.7 (0.7–35.0) pmol/L, *p* = 0.03]. There was no significant difference in LVEF between patients with and without cGVHD.

**Fig. 2:**
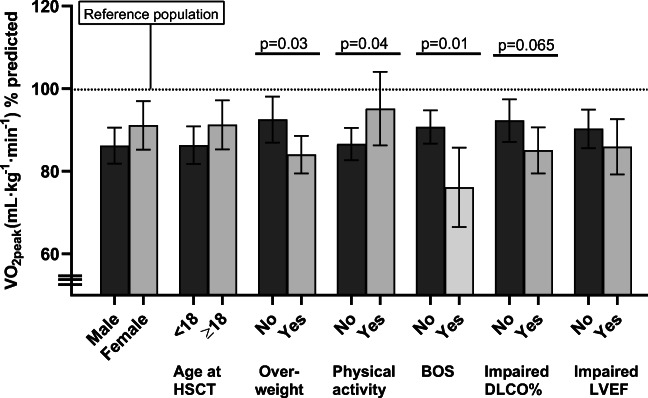
The main results from CPET showing V̇O2peak % predicted (mean 95 % CI) and key variables

The main results from CPET are presented in Fig. 2. For the entire study group, V̇O_2_peak was mean (95% CI) 36.4 (34.7–38.0) mL kg^−1^ min^−1^, corresponding to 89 (85–93)% of the predicted value. Low cardiorespiratory fitness was found in 39 (43%) of the subjects. Survivors with BMI ≥ 25 had lower V̇O_2_peak % predicted than those with normal weight [84% (79–89) vs 92% (87–98), *p* = 0.03]. Only 29% of the survivors met the WHO recommendations for physical activity. Those who met the recommendations had higher mean V̇O_2_peak % predicted than those who did not [95 (86–104) vs 86 (82–90), *p* = 0.04]. Survivors with BOS had reduced V̇O_2_peak % predicted compared with those without BOS [76 (66–86) vs 91 (87–95), *p* = 0.01]. Those with BOS also had reduced breathing reserve (*p* = 0.002) and HR reserve (*p* = 0.01). There was a positive correlation between V̇O_2_peak % predicted and, respectively, LVEF and DL_CO_ % predicted (*p* = 0.04 and 0.06).

Detailed data on cardiorespiratory responses during CPET are presented in Supplement 1. All subjects were able to obtain peak effort (Borg scale ≥ 18 and/or RER ≥ 1.10). Of the 39 (43%) subjects with low cardiorespiratory fitness, 22 (56%) were deconditioned, while 17 (44%) had either cardiac (*n* = 6), lung volume (*n* = 6), gas exchange (*n* = 2), or several limiting factors (*n* = 3). In a multiple linear regression model, low V̇O_2_peak % predicted was significantly associated with high BMI, not meeting WHO recommendations for physical activity, BOS, low DL_CO_ % predicted, and reduced LVEF (Table 3).

**Table 3:** Univariate and multiple regression analysis using V̇O_2_peak % of predicted as dependent variable in long-term survivors of allo-HSCT (*n* = 90)

Variable	Unadjusted (ß)	95% CI	*p* value	Adjusted (ß)^1^	95% CI	*p* value
Female gender	5	(− 2.6, 12.6)	0.2			
BMI	− 1.1	(− 1.8, − 0.4)	0.002	− 1.4	(− 2.0, − 0.8)	< 0.001
Age at allo-HSCT	0.2	(− 0.2, 0.6)	0.26			
Chronic GVHD w/o BOS	− 2.5	(− 10.8, 5.8)	0.55			
BOS	− 14.6	(− 25.8, − 3.4)	0.01	− 15.2	(− 24.9, − 5.4)	0.003
Physical activity	8.8	(0.6, 17.0)	0.04	7.3	(0.2, 14.4)	0.04
DL_CO_ % of predicted	0.2	(− 0.01, 0.5)	0.06	0.2	(0.04, 0.5)	0.02
LVEF	0.6	(− 0.03, 1.3)	0.06	0.6	(0.07, 1.2)	0.03

^1^Adjusted for all shown variables. *Allo-HSCT*, Allogeneic hematopoietic stem cell transplantation; *BOS*, bronchiolitis obliterans syndrome; *BMI*, body mass index; *GVHD*, graft-versus-host disease; *DL*_*CO*_, diffusing capacity for carbon monoxide; physical activity according to the World Health Organization recommendation; *LVEF*, left ventricular ejection fraction

## Discussion

The study demonstrated that median 17 years after allo-HSCT, only half of the survivors had normal cardiorespiratory fitness assessed by V̇O_2_peak. Among the survivors with low cardiorespiratory fitness, both cardiopulmonary factors and deconditioning were found to limit exercise and were equally common. Low cardiorespiratory fitness was associated with reduced gas diffusion capacity, reduced systolic ventricular function, BOS, and overweight and inactivity. In sum, these findings encourage long-term monitoring of survivors after allo-HSCT and highlight the need for increased focus on modifiable lifestyle factors in oncology survivorship clinics.

Several previous studies have reported reduced cardiorespiratory fitness after allo-HSCT [10–14]. However, nearly all those studies were performed in children and adolescents [11–14]. Eames et al. estimated that as much as 68% of their cohort had V̇O_2_peak < 80% predicted [12]. Larsen et al. [11] and Hogarty et al. [13] found impaired cardiorespiratory fitness with reduced V̇O_2_peak in 55% and 69% of the patients, respectively. Those studies all had small sample sizes (< 40), comprised heterogeneous groups including patients with both blood disorders and lymphomas, TBI conditioning, and mean follow-up was < 7 years. One larger study including young patients (mean age 14 years) with blood disorders only reported reduced V̇O_2_peak in 25% of 63 survivors with mean observation 7 years [14]. To our knowledge, only one previous long-term study has been performed in adults [10]. That study was confined to an older population (median age 67 years), but included only 20 subjects (10 allo-HSCT and 10 auto-HSCT), and found, on average, 22% lower V̇O_2_peak in the survivors as compared with predicted. None of the studies above is directly comparable to ours since we studied very long-term effects of allo-HSCT in young adults conditioned with chemotherapy only.

We found that mean V̇O_2_peak was 89% of predicted, adjusted for gender and age. This is considerably higher than in the studies above. Furthermore, none of our survivors had V̇O_2_peak < 16 mL kg^−1^ min^−1^, a threshold reported to be associated with a 9-fold risk of death in allo-HSCT patients [32]. Given the other studies, all had much shorter observation periods than ours, there is a possibility that V̇O_2_peak may improve over time. Survivorship bias is another potential explanation, suggesting that very long-term allo-HSCT survivors eligible for CPET comprise a selected group who has been spared serious complications. Another factor is the mode of exercise testing. The survivors in our study were tested on a treadmill while in the other studies—except one [12]—the subjects were tested on a cycle ergometer. Walking on a treadmill is a more functional way of moving than cycling; it places a higher demand on the cardiorespiratory system and is known to elicit 10–20% higher V̇O_2_peak because larger muscle groups are involved [29]. Finally, in contrast to the other mentioned studies, the survivors in our study had not been treated with TBI that may cause both cardiac and pulmonary injury [33].

About half of the survivors with low cardiorespiratory fitness were deconditioned. Deconditioning is closely linked to modifiable lifestyle factors such as overweight and inactivity. More than 70% of the survivors did not meet the WHO recommendations for physical activity, and 42% were overweight. Those with overweight had features of metabolic syndrome (hypertension, dyslipidemia). A report from the St. Jude Lifetime Cohort Study concluded that long-term adult survivors of childhood and adolescent malignancies were more likely to have metabolic syndrome with > 2-fold relative risk if they did not follow physical activity and diet guidelines set out by the World Cancer Research Fund and American Institute for Cancer [34]. The metabolic syndrome is associated with cardiovascular death and increased mortality by all causes, and even low levels of physical activity may reduce the risk [35].

Cardiovascular disease has been shown to be a leading cause of late morbidity and mortality after allo-HSCT [2] and survivors of allo-HSCT have a 4-fold higher risk of developing cardiovascular disease when compared with the general population [6]. A report from the Blood and Marrow Transplant Survivor Study-2 provided data on cause-specific late mortality after allo-HSCT [36]. In a large cohort (*n* = 764), the most common causes of death among those surviving > 10 years were secondary malignancies (36%), infection (22%), and cardiac disease (14%).

Late adverse effects of allo-HSCT on cardiac function have been reported in several studies [5, 6, 37, 38]. In a recent study, Vandekerckhove et al. found echocardiographic signs of systolic and diastolic dysfunction as well as reduced cardiorespiratory fitness in a cohort of 43 children (mean age 13.6 years, mean 6.6 years after allo-HSCT) compared with healthy controls [39]. In our study, none of the survivors had signs or symptoms of manifest left ventricular dysfunction at rest, and for the total study group, mean LVEF was normal. One-third of the subjects had systolic dysfunction, but only two subjects had LVEF < 40%. We found that LVEF was significantly associated with V̇O_2_peak % predicted, suggesting that subclinical left ventricular dysfunction may have contributed to low cardiorespiratory fitness.

## Strengths and limitations

The main strengths of the present study are a homogeneous national cohort, very long-term follow-up, and comprehensive cardiopulmonary evaluation with methods that permit detecting key organ-specific impairments. A weakness is the lack of pre-treatment data, which prevents the analysis of longitudinal changes. Since age at diagnosis ranged from 0.3 to 30 years, it might have been possible to obtain reliable baseline CPET results from a subset of the patients. The survivors are young adults and longitudinal data are needed to determine if their cardiorespiratory fitness will improve, deteriorate, or remain stable throughout their adulthood. Furthermore, the cross-sectional design does not permit us to define causal relationships. Although we consider an attendance rate of 66% to be acceptable in a very long-term follow-up study, we cannot rule out that non-response bias has affected the external validity and generalizability of our results since the non-responders were somewhat younger than the participants and comprised more males. However, our non-response findings mirror those reported in a recent nationwide Norwegian study of young cancer survivors that found no evidence of non-response bias [40].

## Conclusions and recommendations

Half of the survivors had reduced cardiorespiratory fitness median 17 years after allo-HSCT without TBI conditioning. Cardiopulmonary factors and deconditioning were equally common limitations to exercise. We recommend targeted advice on modifiable lifestyle factors and encourage allo-HSCT survivors to follow guidelines for physical activity and diet set out by national and international cancer federations. Life-long alertness for the prevention and detection of cardiopulmonary late effects is also recommended for this increasing population of vulnerable long-term allo-HSCT survivors.

## Electronic supplementary material

ESM 1 (DOCX 14 kb)

## Data Availability

The datasets generated during and/or analyzed during the current study are available from the corresponding author on reasonable request.
