# Supplementary material for: Determinants of cardiorespiratory fitness in very long-term survivors of allogeneic hematopoietic stem cell transplantation: a national cohort study
Source: Support Care Cancer. 2020 Aug 21;29(4):1959–67. doi: 10.1007/s00520-020-05644-1 (PMC7892519; doi:10.1007/s00520-020-05644-1)
Supplement: Supplementary file 1 — (DOCX 14 kb) [file 520_2020_5644_MOESM1_ESM.docx]

| **Supplement 1:** Results from the cardiopulmonary exercise test of 90 long-term survivors of allo-HSCT divided into normal (V̇O_2_ ≥ 85 % of predicted) vs impaired (V̇O_2_ < 85% of predicted) | | | | |
| --- | --- | --- | --- | --- |
|  | **All** | **Normal** | **Low** |  |
|  | **n=90** | **n=51** | **n=39** | **p-value** |
|  | **(100%)** | **(57%)** | **(43%)** |  |
| V̇O_2_ peak, L·min^-1^ | 2.58 (2.43-2.72) | 2.72 (2.53-2.92) | 2.39 (2.20-2.59) | N/A |
| V̇O_2_ peak, mL·kg^-1^·min^-1^ | 36.4 (34.7-38.4) | 39.5 (37.6-41.3) | 32.3 (29.9-34.7) | N/A |
| V̇O_2_ peak, % of predicted | 89 (85-93) | 101 (97-105) | 73 (71-76) | N/A |
| VE, L·min^-1^ | 97 (91-103) | 106 (98-113) | 85 (77-94) | **<0.001** |
| BR, % from MVV | 23 (20-26) | 21 (18-25) | 25 (21-30) | 0.17 |
| BR < 15 % or 11 L·min^-1^, n | 19 (21 %) | 12 (24 %) | 7 (18 %) | 0.52 |
| VE/V̇CO_2_ slope | 28.1 (26.7-29.5) | 29.6 (27.5-31.6) | 26.1 (24.3-27.8) | **0.015** |
| VE/V̇CO_2_ slope > 34, n | 11 (18 %) | 8 (16 %) | 3 (8 %) | 0.33 |
| HR_max_, beats/min | 182 (179-185) | 181 (178-185) | 183 (178-189) | 0.46 |
| HR_max_, % of predicted | 95 (92-98) | 98 (97-100) | 91 (83-98) | **0.03** |
| O_2_ pulse, mL/beat | 14.3 (13.5-15.1) | 15.3 (14.1-16.4) | 12.9 (11.9-13.9) | **0.004** |
| O_2_ pulse, % of predicted | 89 (85-93) | 97 (92-102) | 78 (72-84) | **<0.001** |
| RER, VCO_2_/VO_2_ | 1.18 (1.16-1.20) | 1.20 (1.18-1.22) | 1.16 (1.13-1.19) | **0.03** |
| Allogeneic hematopoietic stem cell transplantation; allo-HSCT, Maximum oxygen uptake; V̇O_2_, bronchiolitis obliterans syndrome; BOS, ventilation; VE, maximum minute ventilation; BR, breathing reserve; MVV, ventilatory equivalent for CO_2_; VE/V̇CO_2_, heart rate; HR, respiratory exchange ratio; RER, not applicable; N/A. Comparison between Normal and Impaired. Data presented as mean (95% Confidence interval) or number (%). | | | | |

**Article title:** Determinants of cardiorespiratory fitness in very long-term survivors of allogeneic hematopoietic stem cell transplantation: A national cohort study.

**Journal name:** Journal of Supportive Care in Cancer

**Author list:** Ole Henrik Myrdal, Phoi Phoi Diep, Ellen Ruud, Lorentz Brinch, Richard John Massey, Elisabeth Edvardsen, Johny Kongerud, May B. Lund and Liv Ingunn Sikkeland.

**Corresponding author:** Ole Henrik Myrdal, Dept of Respiratory Medicine, Oslo University Hospital, Rikshospitalet, Norway, Box 4950 Nydalen, 0424 OSLO, Norway
Phone: +47 23072808, Fax: +47 23073917: E-mail: omyrda@ous-hf.no
